# The influence of supraliminal priming on energy density of food selection: a randomised control trial

**DOI:** 10.1186/s40359-021-00554-1

**Published:** 2021-03-23

**Authors:** Isabelle Schlegel, Sharon A. Carstairs, Gozde Ozakinci

**Affiliations:** 1 School of Medicine, University of St Andrews, North Haugh, St Andrews, KY16 9TF UK; 2 Present Address: School of Medicine, University of Glasgow, Gilbert Scott Building, University Ave, Glasgow, G12 8QQ UK

**Keywords:** Compensatory eating, Energy density, Exercise, Priming, Visual cues

## Abstract

**Background:**

Many people exercise because they know it is good for their health. Although this is true, it can make us feel deserving of a reward and lead us to eat more indulgent, less healthy food than if we had not done any exercise. Generally, lower energy-dense (LED) foods are recognised as healthier choices than higher energy-dense (HED) options. Despite our intention to make healthy choices, seeing tempting higher-calorie foods on offer often side-tracks us. Priming is a psychological tool that makes specific changes to our environment that remind us of our motivation to be healthy. This makes it easier to choose a healthier option, by nudging us towards it without us even realising. However, it is currently unclear which method of priming achieves the best results.

**Aims:**

Our study explores whether priming people to expect they will receive LED food leads them to make this healthier choice after exercise, even when also offered tempting less healthy HED foods at the moment of selection.

**Methods:**

Our study observed the foods selected by university athletes after their sports matches. Before the match, half of the participants were primed by asking them to choose a LED snack from the options we offered, which they would receive after the match. The remaining half of participants were not asked this same question. To distract the athletes from our observation of their food choices, participants completed a task prior to choosing their snack, which was disguised as a ‘thank you’ for taking part.

**Results:**

Overall, we found the priming group did not choose LED foods significantly more than the control group, hence priming did not increase LED food selection.

**Conclusion:**

Importantly, our results indicate that priming must be more noticeable to achieve its goal. Additionally, we demonstrated that priming may be less successful for young athletic individuals, compared to older and more overweight adults recruited in other studies. This highlights the importance of studying a broader demographic range of individuals from the general population. We support future research into this area, which will help us to tweak priming to achieve the best outcomes.

***Trial registration*:**

ISRCTN Registry, ISRCTN74601698. Date registered: 02/10/2020 (retrospectively registered).

**Supplementary Information:**

The online version contains supplementary material available at 10.1186/s40359-021-00554-1.

## Introduction

### Background

The consumption of calorically dense foods post-exercise is commonly observed amongst recreational exercisers, despite their awareness that a lighter option would likely be a healthier choice [1]. Supraliminal priming reduces the amount of effortful self-regulation required to enact healthful behaviours, by presenting individuals with a stimulus designed to directionally alter their behaviour without their conscious awareness [2].

Both evolutionary and socioenvironmental cues have led humans to associate food with deserving a reward in return for expending effort; even the mere thought of exercise can increase caloric consumption [3]. The indulgence in tempting (often calorically denser) food following exercise is explained by multiple theories.

The Compensatory Health Beliefs model proposes that completing one health-promoting behaviour (such as exercise) cognitively “negates” [4] the negative effect of a behaviour that does not support progress towards health goals, for example eating ‘junk food’ [5]. Additionally, the Goal Conflict model illustrates the obstacle of time preference [6]: resisting an immediate short-term hedonic experience in pursuit of a longer-term goal.

Priming can promote behavioural modification following exercise to support longer-term health goals [7], bypassing this conflict of desires. The acutely taxing nature of exercise can lessen our perceived ability to exert self-control, thus rendering us more vulnerable to tempting visual food cues. This tendency is compounded by our evolved genetic tendency to select calorically dense foods.

Previous studies adopting priming interventions to promote healthful food selection used health-promoting vocabulary (such as “wellbeing”) [8, p98, 9,10], traffic light colour schemes or presented images of ‘healthy’ foods (such as fresh vegetables) [7]. However, although promising evidence of priming’s efficacy exists, a delicate balance must be struck to avoid counter-productive outcomes. For example, as predicted by the compensatory health beliefs model, meals explicitly labelled as “low calorie” [5] may be consumed in greater quantities or increase later snack consumption, thus not reducing overall caloric intake, rendering the intervention futile.

### The current study

This study investigates both priming and the effect of exercise on eating behaviour. We recruited participants from a younger, physically active sample because the ‘match tea’ tradition (the organised provision of food following a sports match), is an optimal example of post-exercise hedonic food consumption. However, this phenomenon occurs population-wide, thus the proof of concept derived from this study is widely applicable. Below is the research question this study aimed to answer.

To what extent does priming individuals to select low energy-dense (LED) foods reduce their selection of high energy-dense (HED) items when faced with the temptation of more calorically dense visual cues?

We hypothesised that a greater proportion of the experimental group would select LED foods compared to the control group. The aims of this experimental study, therefore, are two-fold: Firstly, to examine if primed participants switch from their initial LED food choice to a HED item when presented with visual food cues of both options after exercise. Secondly, to observe any differences between the proportion of people selecting LED versus HED foods in control versus experimental groups, to ascertain whether priming efficaciously encourages selection of low energy-dense choices.

## Methods

### Participants

Given this study examines a novel primary outcome measure (food selection in a ‘match tea’ environment), there was a lack of prior studies investigating this primary outcome from which to derive a power calculation for target sample size. However, based on sample sizes of studies adopting similar methodological styles (ranging between 10, 14, 24, 84 and 256 participants) [11–14], 120 participants was estimated to be an appropriate target. This approximately equates to eight 11-person sports teams (plus substitutes). All participants were recruited exclusively from the university’s male and female, 1st and 2nd football and hockey teams. In total 128 participants were recruited for this study by the researchers. Recruitment took place between 27th January 2020 and 6th February 2020, whilst participant involvement and follow-up spanned from 29th January 2020 to 12th February 2020. The study ended upon completion of data collection at all eight sports matches.

### Study design

Using Microsoft Excel stratified random computer-generated allocation, the researchers allocated one male and one female team from each sport to the control and experimental groups, thus conducting randomisation at a group level. To minimise demographic variation between control and experimental groups, randomisation allocation restrictions ensured that both groups contained: one female football, one male football, one female hockey and one male hockey team. This deliberate allocation of equal numbers of male and female teams to each group aimed to reduce the impact of gender on food choice. This was a single-blinded study, given the researchers performed participant randomisation into control and experimental groups and were thus aware of their allocations to ensure the correct pre-match questionnaire was given to each group (allocation ratio 1:1) but this was concealed from participants, to hide the presence of the priming intervention.

To disguise our focus on food-related behaviour, all questionnaires included ‘filler’ questions such as “How often do you sleep more than 8 h in the night?". Questions covered topics including sleep, exercise, social activities and academic work.

Our study design disguised our observation of food selection using a distractor task; the success of our manipulation was reflected by participant questionnaire responses. Only three (of 107) respondents listed ‘eating habits’ as their perceived focus of our study, whilst the majority responded ‘exertion’. This study was retrospectively registered under the ISRCTN Registry, ISRCTN74601698 https://doi.org/10.1186/ISRCTN74601698 and fulfils the CONSORT guideline criteria (evidence of satisfying these criteria can be found in ‘Additional file 6—CONSORT 2010 Checklist’); there was no deviation from this protocol.

### Experimental group

All participants were asked to complete three questionnaires;—baseline, pre-match and post-match (detailed in *Measures*). The baseline questionnaire was completed online by participants 1 week prior to the sports match. The pre-match questionnaire was completed by participants in the changing room on match day, immediately before their sports match. Only the experimental group received a priming intervention; the final question of the pre-match questionnaire asked them to select a LED food item for post-match consumption (whereas this question was replaced by a filler question for the control group).

The post-match questionnaire was completed immediately after the match, upon participants returning to the changing room. Following post-match questionnaire completion, all participants were offered a single snack of their choice, from three LED (apple, banana, orange) and three HED (Galaxy chocolate bar, Belvita granola bar and KitKat biscuit) options. The item selected was the primary outcome measure, recorded by the researcher present. The cut-off between energy density categories was 2.5 kcal/g [15], operationalised to represent ‘healthy’ (LED) and ‘less healthy’ (HED) choices. Ingredients lists of processed foods were displayed and individuals with food allergies were advised to consider this risk before participating. Therefore, the offering of both LED and HED foods post-match surprised the experimental group participants, given the priming intervention had led them to expect to only have LED foods on offer. Whether experimental group participants stuck to a LED food or switched to a HED option was of particular interest and indicated whether the priming intervention significantly nudged individuals towards healthier food choices.

### Control group

The control group completed the baseline, pre-match and post-match questionnaires adhering to the same protocol as the experimental group. However, the control group pre-match questionnaire did not include the priming intervention question; this question was replaced by a filler question. Control group participants also completed the same distractor task and selected a food from the same selection of snacks provided the experimental group following post-match questionnaire completion.

### Measures

*‘Baseline’ (1 week before primary data collection)* In addition to questions written specifically for this study, the External Eating questions of the DEBQ were integrated. The DEBQ measures three dimensions of eating behaviour: restrained (10 questions), emotional (13 questions) and external eating (10 questions). Participants rate the frequency with which they engage in this behaviour as ‘never’, ‘seldom’, ‘sometimes’, ‘often’ or ‘very often’. The final score for each section is the mean of the scores within that section. The external eating portion of the DEBQ examines participants’ self-reported perception of sensory food cue influence on their eating behaviour, which was of particular relevance to this study given its adoption of external cues (priming) to encourage healthier food choices. Additionally, we asked ‘What food or snack would you usually eat shortly after a match?’ to establish popular snacks amongst participants, ensuring we purchased familiar foods (please see ‘Additional file 2—Baseline Questionnaire’).

*‘Pre-match’ (immediately before the sports match)* In this questionnaire, only the experimental group were told about post-match food provided as a ‘thank you’ and asked to choose one of three LED options (HED snacks were not mentioned)—this was the priming intervention (please see ‘Additional file 3—Control Pre-match Questionnaire’ and ‘Additional file 4—Experimental Pre-match Questionnaire’).

*‘Post-match’ (immediately after the sports match)* This written questionnaire was completed in the sports centre changing rooms. It was then followed by a task entailing evaluation of team sport photographs, to disguise our observation of participants’ food item selection. Participants were told this final task must be completed alone, hence facilitating isolation of a participant from team members, to remove social influences on food selection (please see ‘Additional file 5—Post-match Questionnaire’).

Questions related to frequency (such as “How often do you exercise when you feel mentally exhausted?") were either open-ended or asked participants to indicate which of the following applied: never, seldom, sometimes, often or very often. This scale was adopted from the Dutch Eating Behaviour Questionnaire (DEBQ) [16], to avoid drawing attention to the External Eating questions integrated into our questionnaires. Subjective appetite was the secondary outcome measure, rated by all participants on a 0–10 visual analogue scale in pre-match and post-match questionnaires.

### Data analysis

**Fig. 1:**
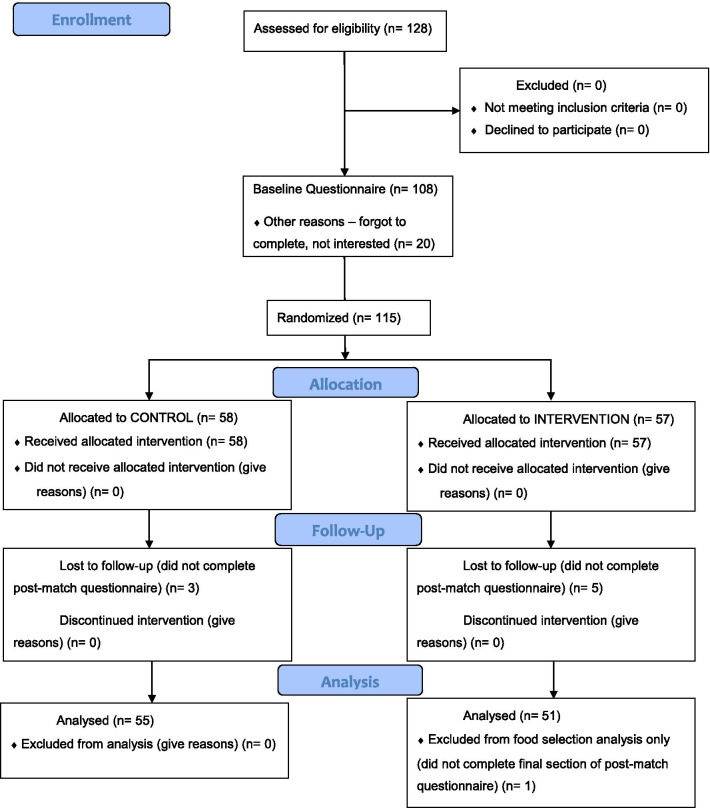
CONSORT 2010 flow diagram

Participant recruitment, randomisation, follow-up and data analysis is illustrated below in ‘Fig. 1—CONSORT 2010 Flow Diagram’. The baseline questionnaire was completed by participants (n = 108) before randomization took place on match day, including by some individuals who were not selected for the match by the coaches, hence these participants did not go on to complete pre and post-match questionnaires (n = 13). Some participants (n = 20) completed the pre- and post-match (but not the baseline) questionnaires; these 33 participants were excluded from analyses. Of the 107 participants completing all three questionnaires, one individual did not complete the final section of the post-match questionnaire in which participants entered the study room one by one; therefore, they did not have the opportunity to select a food item, so analyses involving food item selection totalled n = 106 (whilst analyses for all other measures totalled n = 107).

Participant attrition was only 8% and the reason for loss to follow-up was most likely these participants’ lack of time to complete a questionnaire after their match. The time taken to participate was identical for control and experimental groups because they were asked to complete the same questionnaires (except for one question being different in the pre-match questionnaire). Therefore, given the reason for participant attrition was most likely the same amongst both groups and this loss did not cause the groups’ demographics to notedly differ from one another, the missing data was ignorable and data imputation was not deemed necessary.

All data was de-identified by allocating each participant a number (simply in order of data collection). Data relevant for statistical analysis was coded into numerical format, before it was inputted into IBM SPSS Statistics 25 [17]. The energy density of food selected post-match was coded as follows: LED = 1, HED = 2. Additionally (applicable to experimental group participants only), sticking to a LED food = 1, whereas switching their choice to a HED item = 2. It must be noted that participants changing their selection to a different LED item to the one initially chosen on their pre-match questionnaire were not categorised as making a ‘switch’ because all LED items were fruits of similar absolute caloric value. Hypotheses were stated before data collection and the data analysis plan was pre-specified.

Pearson Chi-square tests were performed to compare LED versus HED food selection between control and experimental groups. The effect size was measured using Phi’s coefficient (Φ) using the parameters: small (0.1 < *Φ*), medium (0.3 < *Φ*) and large (0.5 < *Φ*) (43); *p* < 0.05 (95% confidence interval) was used to determine the statistical significance of results. A frequency calculation was used to establish the proportion of experimental group participants who had selected an LED or HED item.

This study’s secondary outcome measure explored the effect of exercise on appetite. A paired t-test was performed to ascertain whether self-reported appetite had significantly changed following exercise, by comparing mean cohort (combined and experimental group) values before and after the match. Additionally, a Pearson Chi-square test examined whether participants with a higher post-match appetite were more likely to select HED foods. The median appetite rating formed the boundary between ‘low appetite’ and ‘high appetite’ groups for the purpose of this calculation.

We had no hypothesis predicting the proportion of participants that would select LED or HED items, given the lack of prior studies investigating this phenomenon in relation to priming. One participant excluded from food selection analysis was a member of the experimental group, thus analysis of this group in isolation had 51 participants.

We predicted that primed participants would be less influenced by tempting HED visual food cues because they expected to receive their LED food of choice. The control group had not made this same initial LED item selection and so were not similarly in the mindset of thinking that they would be receiving a LED food to eat post-match.

We hypothesised that self-reported appetite would be lower after the match than it was before, given the strong literature supporting the exercise-induced anorexia phenomenon [13]. Additionally, we predicted a stronger post-match appetite to increase an individual’s tendency to select HED food, but that priming could minimise this phenomenon by instilling an expectation of consuming a LED item.

## Results

### Experimental group

The experimental group initially contained 29 female and 28 male participants (with the same 18–23-year-old age range), which became 28 females and 23 males for analyses.

### Control group

The control group consisted of 58 participants, including 28 female and 30 male athletes. The age range was 18–23 years old. Following participant attrition, analyses for this group comprised 26 female and 29 male participants.

### Quantitative results

Analyses showed 37.3% of primed participants chose a LED item, sticking to a LED choice (Table 1). Table 1 illustrates the data collected; there was no significant association between participant randomisation groups and whether an LED or HED food was selected Χ^2^(1) = 0.797, *p* = 0.372 (95% confidence interval). Please see ‘Additional file 1—Dataset’ (a Microsoft Excel document) for all data collected, both in its raw and numerically coded forms. Although effect sizes were calculated, given the lack of significant results these were not deemed relevant to include in the table below.

**Table 1:** Frequencies of LED/HED food selection by randomised participant groups (N = 106)

Randomised group	LED		HED	
	N	%	N	%
Control	16.0	29.1	39.0	70.9
Experimental	19.0	37.3	32.0	62.7

Contrastingly, on average self-reported post-match appetite (M = 5.170, SE = 2.704) was higher than pre-match appetite (M = 3.030, SE = 2.094). This difference, 2.140, 95% CI [11.602, 2.679], was significant, *t*(106) = 7.879, *p* < 0.001. Additionally, there was no significant association between post-match appetite and food selection, hence the null hypothesis was accepted *X*^2^(1) = 0.114, *p* = 0.735.

## Discussion

The coupling of sedentary lifestyles with constant access to surplus food have created an obesogenic environment [18, 19], facilitating excessive hedonic food consumption and subsequent weight gain. Although information provision about healthy eating is essential, in isolation this is often insufficient to instigate behavioural change, whereas priming can make this easier by lessening the degree of self-control required to make healthier choices.

### Study findings

We found 62.7% of experimental participants switched from a LED to a HED item, indicating our prime lacked significant efficacy. This suggests tempting HED visual food cues influenced food selection more powerfully than our priming intervention. Priming does have the ability to evoke behavioural change; however, its efficacy is highly dependent on its format and the sample demographics [8, 20, 21].

Studies achieving significant efficacy with priming interventions [5, 7, 20, 21] adopted health-promoting visual cues and vocabulary, whereas our priming intervention simply primed experimental group participants to expect they would be receiving a LED food. Our subtler priming strategy purposefully did not refer to the lower calorie content of LED foods, to avoid framing these as less satisfying than HED options. However, it appears this mere expectation of consuming an LED food too subtle a prime to efficaciously encourage actual LED food selection.

Although we did not quantitatively measure time taken to select a food, we observed that primed participants did take noticeably longer. This prolonged decision-making time did not always translate into choosing a LED item, but perhaps the expectation of receiving a ‘healthy’ snack led individuals to think more carefully about the healthfulness of the foods on offer, rather than solely their palatability. Moreover, Bargh et al. [22] proposed that priming’s efficacy greatly decays over time and is optimised by prolonging the exposure to priming, whilst Minas et al. [7] also encourage minimising the delay between exposure and executing the desired behaviour.

### Previous priming studies

However, the heterogeneity of existing studies produces insufficient evidence to substantiate these theories [5, 8], highlighting an area for future research. Prior studies range in their length of exposure to priming, from less than 20 s to more than 10 min, whilst many studies fail to specify [7, 8, 10]. Our prime was only one short question and involved a 3-h delay between exposure and food selection. The combined effect of priming very subtly and briefly, with a considerable delay, may have rendered our priming intervention insufficiently strong to influence food selection.

Although the format of priming greatly impacts its efficacy, numerous additional factors account for inter-individual variation in responsiveness to this intervention. For example, Boland et al. [20] found priming was only efficacious in the afternoon, whereas in the morning no significant difference was found. Given our study’s data collection took place within a 4-h window in the afternoon, the time of day was unlikely to have a bearing on our findings.

Moreover, an individual’s personal health goals greatly influence their response to priming. A meta-analysis conducted by Buckland et al. [23] found healthy eating primes encouraged ‘healthy’ eating behaviour for participants strongly motivated to lose weight, whereas there was no significant effect for those with lesser motivation to achieve this goal. Our sample’s demographic characteristics may have contributed to our non-significant findings, since participants were less likely to have the weight-control motivation to select LED foods. Questionnaire responses regarding motivation to exercise were most frequently for ‘fun’ and ‘social’ purposes, with no participants listing weight-control as a reason. Given the selection bias of our sample, our findings are not to be extrapolated to the general population and highlight that priming’s efficacy is affected by its sample demographics.

Paradoxically, priming may be least efficacious for those who would profit from it most: those with less dietary restraint and thus more likely to benefit from making more healthful food choices or losing weight [8]. Buckland et al. [21] found priming women with “diet-congruent” (LED) versus “diet-incongruent” (HED) foods reduced caloric intake for restrained, but not unrestrained eaters (as measured by the DEBQ). Although our study only utilised the external eating portion of the DEBQ to keep our questionnaire concise, future studies may wish to examine the relationship between priming and all three domains.

### Self-reported appetite

We found a statistically significant increase in self-reported appetite after exercise. This disagreement with our hypothesis may be partially attributable to the debatable influence of exercise-induced anorexia; Pomerleau et al. [24] found this only occurs after exercise of a sufficiently high intensity, whilst King et al. [25] suggest exercise fails to trigger this phenomenon entirely.

Our hypothesis was also influenced by the dehydration-anorexia model [26] predicting appetite reduction after immediately exercise; Pérez-Luco et al. [27] found energy intake significantly reduced amongst post-exercise hypo-hydrated versus rehydrated participants. Additionally, exercise may suppress appetite by increasing activation of neural mesolimbic reward pathways by low-calorie foods, whilst decreasing activation by more hedonic HED foods [28, 29].

Caution is required when comparing the findings of studies using these different measures, given the discrepancy between quantifying appetite using physiological measurements and self-reported subjective appetite ratings; only the latter incorporates the considerable psychological modulators of appetite. Our hedonic desire to eat does not reflect our homeostatic caloric requirements, hence our eating behaviour often fails to reflect physiological measures of appetite. This incongruity contributes to explaining why our results did not support our hypothesis, since we did not measure physiological appetite.

Moreover, we hypothesised that individuals with a greater self-reported post-match appetite would select HED foods more frequently than those with a lower appetite; however, our data exhibited no significant effect of appetite on food choice. This may be partly attributable to the aforementioned limitations of self-reporting. A systematic review conducted by Holt et al. [30] supports this reasoning, finding only 51% of 462 studies analysed exhibited a link between self-reported appetite and energy intake. A further consideration is the dietician’s nutritional advice these student athletes receive. Following this guidance, they may eat more independently of appetite cues [31], for example intentionally eating more calories to replenish energy stores.

### Strengths and limitations

It must be noted that the lack of a significant effect may be attributable to the inadequate sample size as we were not able to do a priori sample size calculation, rather than absence of actual effect. All food items were sweet, to eliminate the confounding effect sweet versus savoury preference on food selection. Four pieces of each food were always laid out identically, so the visual food cue was the same for each participant. Time pressure greatly influences food choices, particularly amongst university athletes [32], hence all foods required no cooking time.

However, there was inter-individual variation in energy expenditure; for some athletes, HED food selection was not ‘less healthy’, rather may have been an appropriate choice to restore energy balance. We did not record how much of the selected food was consumed, to avoid drawing attention to the study focus. Moreover, the food chosen is merely a snapshot; it is not representative of individuals’ overall food consumption from prior and later meals, which ultimately determines whether energy balance is achieved. For data analysis purposes we limited participants to selecting one food item only, whereas they may have selected multiple if given the opportunity.

## Conclusion

In summary, this study found results both novel and relevant to existing priming literature. This was the first study to investigate the efficacy of priming individuals to select ‘healthy’ (LED) options after exercise without using health-promoting imagery or vocabulary. We generated an expectation for individuals that they would have solely LED foods on offer, whereas they were later faced with both LED and ‘less healthy’ HED visual food cues. Given compensatory eating behaviour commonly ensues after exercise, observing post-match food choices proved to be a logistically opportune study setting.

We found less than half of primed participants stuck to an LED food choice, suggesting our prime was too subtle to efficaciously instigate the desired health-promoting behaviour. Additionally, there was no significant increase in the proportion of LED foods selected by primed participants.

Our results highlight that priming’s efficacy depends on the intervention itself and extraneous factors. Priming bypasses individuals’ conscious awareness that the intervention modulates their behaviour, therefore potentially sidestepping the motivational barrier that so commonly obstructs individuals attempting to make healthier choices. Therefore, we strongly advocate for further research into this field, to increase the range of health-promoting strategies we have at our disposal.

## Supplementary Information

**Additional file 1.** Dataset, Dataset, all anonymised raw questionnaire data.

**Additional file 2.** Baseline Questionnaire, Participant Baseline Questionnaire, questionnaire completed by all participants within one week prior to sports match.

**Additional file 3.** Control Pre-match Questionnaire, Control Participant Pre-match Questionnaire, questionnaire completed within 30 minutes of sports match by control group participants.

**Additional file 4.** Experimental Pre-match Questionnaire, Experimental Participant Pre-match Questionnaire, questionnaire completed within 30 minutes of sports match by experimental group participants.

**Additional file 5.** Post-match Questionnaire, Participant Post-match Questionnaire, questionnaire completed by all participants immediately after sports match.

**Additional file 6.** CONSORT 2010 Checklist, CONSORT 2010 Checklist, CONSORT 2010 Checklist in accordance with CONSORT guidelines.

## Data Availability

All questionnaires and data generated or analysed during this study are included in this published article. The dataset supporting the conclusions of this article is included within the article and its additional files.

## Abbreviations

CONSORT: Consolidated Standards of Reporting Trials
DEBQ: Dutch Eating Behaviour Questionnaire
LED: Low energy-dense
HED: High energy-dense
