# Supplementary material for: The influence of supraliminal priming on energy density of food selection: a randomised control trial
Source: BMC Psychol. 2021 Mar 23;9:48. doi: 10.1186/s40359-021-00554-1 (PMC7988930; doi:10.1186/s40359-021-00554-1)
Supplement: Supplementary file 2 — Additional file 2. Baseline Questionnaire, Participant Baseline Questionnaire, questionnaire completed by all participants within one week prior to sports match. [file 40359_2021_554_MOESM2_ESM.pdf]

Participant Baseline Questionnaire

You are being invited to participate in a research study titled: *The Life of an Athlete at University*. This study is being done by Isabelle Schlegel from the University of St Andrews.

The purpose of this research study is to explore the impact of the team sport a student athlete plays (to a competitive level) on their physical, psychological and social wellbeing, including: sleep, nutrition, social life and academic work. This questionnaire will take you approximately 10 minutes to complete.

Your participation in this study is entirely voluntary and you can withdraw at any time. You are free to omit any question.

Full Name: ____

Height (cm or feet/inches): Weight (kg or stones/pounds):

**1. How often do you use protein supplementation, such as protein powder?**				
Never ☐	Seldom ☐	Sometimes☐	Often ☐	Very often☐
**2. If food tastes good to you do you eat more than usual?**				
Never ☐	Seldom ☐	Sometimes☐	Often ☐	Very often☐
**3. How often do you take vitamin or mineral supplements?**				
Never ☐	Seldom ☐	Sometimes☐	Often ☐	Very often☐
**4. How often do you drink more than the national guideline of a maximum of 14 units of alcohol per week?**				
Never ☐	Seldom ☐	Sometimes☐	Often ☐	Very often☐
**5. If you have something delicious to eat do you eat it straight away?**				
Never ☐	Seldom ☐	Sometimes☐	Often ☐	Very often☐
**6. How often do you stretch for at least 5 minutes after a sports match?**				
Never ☐	Seldom ☐	Sometimes☐	Often ☐	Very often☐
**7. How often do you drink beverages other than water before, during or after exercise?**				
Never ☐	Seldom ☐	Sometimes☐	Often ☐	Very often☐
**8. How often do you train in your team sport during the off-season?**				
Never ☐	Seldom ☐	Sometimes☐	Often ☐	Very often☐
**9. If you see others eating, do you also want to eat?**				
Never ☐	Seldom ☐	Sometimes☐	Often ☐	Very often☐
**10. If you see others drinking alcohol, do you also want to drink alcohol?**				
Never ☐	Seldom ☐	Sometimes☐	Often ☐	Very often☐
**11. When preparing a meal are you inclined to eat something?**				
Never ☐	Seldom ☐	Sometimes☐	Often ☐	Very often☐
**12. How frequently do you listen to music or watch videos when exercising independently?**				
Never ☐	Seldom ☐	Sometimes☐	Often ☐	Very often☐
**13. Do you find exercise to be a stress-relieving activity?**				
Never ☐	Seldom ☐	Sometimes☐	Often ☐	Very often☐
**14. When you drink alcohol, how often do you binge drink (consume more than 8/6 units of alcohol in one session for men/women)?**				
Never ☐	Seldom ☐	Sometimes☐	Often ☐	Very often☐

15. **If you see or smell something delicious to eat, do you eat it straight away?**<br>Never ☐ Seldom ☐ Sometimes☐ Often ☐ Very often☐
16. **How often do you socialise with members of your sports team outside training or matches?**<br>Never ☐ Seldom ☐ Sometimes☐ Often ☐ Very often☐
17. **Since joining this sports team at university, have you ever considered giving it up?**<br>Never ☐ Seldom ☐ Sometimes☐ Often ☐ Very often☐
18. **Do you plan to continue playing your sport after you leave university?**<br>Never ☐ Seldom ☐ Sometimes☐ Often ☐ Very often☐
19. **Do you eat more than usual when you see others eating?**<br>Never ☐ Seldom ☐ Sometimes☐ Often ☐ Very often☐
20. **Do you drink more alcohol than usual when you see others drinking alcohol?**<br>Never ☐ Seldom ☐ Sometimes☐ Often ☐ Very often☐
21. **How often do you drink at least the recommended daily water intake (1.2-1.9L)?**<br>Never ☐ Seldom ☐ Sometimes☐ Often ☐ Very often☐
22. **Can you resist eating delicious food?**<br>Never ☐ Seldom ☐ Sometimes☐ Often ☐ Very often☐
23. **Can you resist drinking alcohol on a social occasion?**<br>Never ☐ Seldom ☐ Sometimes☐ Often ☐ Very often☐
24. **How often does a sports injury prevent you from taking part in team training or a match?**<br>Never ☐ Seldom ☐ Sometimes☐ Often ☐ Very often☐
25. **If you walk past the bakery do you have the desire to buy something delicious?**<br>Never ☐ Seldom ☐ Sometimes☐ Often ☐ Very often☐
26. **How often do you sleep more than 8 hours in the night?**<br>Never ☐ Seldom ☐ Sometimes☐ Often ☐ Very often☐
27. **How often do you consider miss a training session or match due to academic workload?**<br>Never ☐ Seldom ☐ Sometimes☐ Often ☐ Very often☐
28. **How often do you miss a training session or match due to academic workload?**<br>Never ☐ Seldom ☐ Sometimes☐ Often ☐ Very often☐
29. **If food smells and looks good, do you eat more than usual?**<br>Never ☐ Seldom ☐ Sometimes☐ Often ☐ Very often☐
30. **How often do you sleep more than 6 hours in the night?**<br>Never ☐ Seldom ☐ Sometimes☐ Often ☐ Very often☐
31. **How often do you exercise the day after consuming alcohol?**<br>Never ☐ Seldom ☐ Sometimes☐ Often ☐ Very often☐
32. **How often do you exercise when you feel mentally exhausted?**<br>Never ☐ Seldom ☐ Sometimes☐ Often ☐ Very often☐
33. **If you walk past a snack bar or a café, do you have the desire to buy something delicious?**<br>Never ☐ Seldom ☐ Sometimes☐ Often ☐ Very often☐
34. **How often do you wake up more than once from sleep during the night?**<br>Never ☐ Seldom ☐ Sometimes☐ Often ☐ Very often☐
35. **How often do you exercise when you feel physically exhausted?**<br>Never ☐ Seldom ☐ Sometimes☐ Often ☐ Very often☐
36. **How often do you exercise to the point of feeling you have reached your physical limit?**<br>Never ☐ Seldom ☐ Sometimes☐ Often ☐ Very often☐

37. **How often do you exercise to the point of feeling you have reached your mental limit?**
Never ☐ Seldom ☐ Sometimes☐ Often ☐ Very often☐

38. **If you see or smell something delicious, do you have a desire to eat it?**
Never ☐ Seldom ☐ Sometimes☐ Often ☐ Very often☐

39. **How many hours do you sleep on average per night?**

40. **How many days in the week do you exercise (includes training, matches, other activities)?**

41. **What food or snack would you usually eat shortly after a match?**

42. **On average, how many units of alcohol do you drink per week? (1 unit = half pint of larger, one 25ml shot of spirits)**

43. **On average, how many days in the week do you feel tired?**

44. **On average, how many days in the week do you spend working on your academic studies for more than six hours?**

45. **How many times have you been ill within the past 12 months (including cold and flu)?**

46. **What is the highest level to which you have played your team sport (e.g. school/university, club, county, national)?**

47. **Do you have any allergies? If so, please specify.**

48. **What is your main motivation to play your team sport for the university team?**

49. **Do you compete in an individual sport, in addition to your team sport? If yes, please state which sport(s).**

50. **Which three words would you use to describe your relationship with your team sport?**

Version 1: 14.11.2019
