# Supplementary material for: The influence of supraliminal priming on energy density of food selection: a randomised control trial
Source: BMC Psychol. 2021 Mar 23;9:48. doi: 10.1186/s40359-021-00554-1 (PMC7988930; doi:10.1186/s40359-021-00554-1)
Supplement: Supplementary file 3 — Additional file 3. Control Pre-match Questionnaire, Control Participant Pre-match Questionnaire, questionnaire completed within 30 minutes of sports match by control group participants. [file 40359_2021_554_MOESM3_ESM.pdf]

Participant Pre-Match Questionnaire

You are being invited to participate in a research study titled: *The life of an athlete at university.* This study is being done by Isabelle Schlegel from the University of St Andrews. The purpose of this research study is to explore the impact of the team sport a student athlete plays (to a competitive level) on their physical, psychological and social wellbeing, including: sleep, nutrition, social life and academic work. This questionnaire will take you approximately 5 minutes to complete. Your participation in this study is entirely voluntary and you can withdraw at any time. You are free to omit any question.

Full Name: ____

1. How tired do you feel currently? (BORG Perceived Exertion Scale: 6 = no exertion 20 = maximal exertion)

   ____

2. How many hours did you sleep last night?

   ____

3. How strong is your appetite currently? Please circle your answer.
   0 = not hungry at all, 10 = I have never been more hungry

0	1	2	3	4	5	6	7	8	9	10

For Questions 4 and 5, tick **all** boxes that apply.

4. What forms of training do you undertake during match season?
   Squad pitch sessions ☐ Squad strength & conditioning sessions ☐
   Strength training (e.g. lifting weights) ☐ Cardiovascular training (e.g. running) ☐
   Cross training ☐ Flexibility training (e.g. yoga) ☐
   Water-based training (e.g. swimming) ☐ None ☐

5. Which emotions have you experienced in the past 24 hours?
   Joy ☐ Fear ☐ Disgust ☐ Anger ☐
   Anticipation ☐ Sadness ☐ Surprise ☐ Trust ☐
   Other (please specify) ☐ ______________________________

For Questions 5-10, please tick only **one** box.

6. How much are you looking forward to **playing** in this sports match?
   Not at all ☐ Somewhat ☐ Moderately ☐ Very much ☐

7. What have you eaten in the last two hours?
   Nothing ☐ Small snack (e.g. piece of fruit) ☐ Larger snack (e.g. toast) ☐ Full meal (e.g. pasta) ☐

8. How much are you looking forward to the **exercise** involved in this sports match?
   Not at all ☐ Somewhat ☐ Moderately ☐ Very much ☐

9. How much water have you drunk today?
   None ☐ 0-0.5L ☐ 0.5-1L ☐ 1L+ ☐

10. How motivated do you feel to win this sports match?
    Not at all ☐ Somewhat ☐ Moderately ☐ Very much ☐
