# Supplementary material for: The influence of supraliminal priming on energy density of food selection: a randomised control trial
Source: BMC Psychol. 2021 Mar 23;9:48. doi: 10.1186/s40359-021-00554-1 (PMC7988930; doi:10.1186/s40359-021-00554-1)
Supplement: Supplementary file 4 — Additional file 4. Experimental Pre-match Questionnaire, Experimental Participant Pre-match Questionnaire, questionnaire completed within 30 minutes of sports match by experimental group participants. [file 40359_2021_554_MOESM4_ESM.pdf]

# Participant Pre-Match Questionnaire

You are being invited to participate in a research study titled: *The Life of an Athlete at University***.** This study is being done by Isabelle Schlegel from the University of St Andrews. The purpose of this research study is to explore the impact of the team sport a student athlete plays (to a competitive level) on their physical, psychological and social wellbeing, including: sleep, nutrition, social life and academic work. This questionnaire will take you approximately 5 minutes to complete. Your participation in this study is entirely voluntary and you can withdraw at any time. You are free to omit any question.

**Full Name:** ______________________________

1. How tired do you feel currently? (BORG Perceived Exertion Scale: 6 = no exertion 20 = maximal exertion)

   ______________________________

2. How strong is your appetite currently? Please circle your answer. 0 = not hungry at all, 10 = I have never been more hungry

| | | | | | | | | | | |
|---|---|---|---|---|---|---|---|---|---|---|
| 0 | 1 | 2 | 3 | 4 | 5 | 6 | 7 | 8 | 9 | 10 |

For Questions 3 and 4, tick **all** boxes that apply.

3. What forms of training do you undertake during match season?
   Squad pitch sessions ☐
   Squad strength & conditioning sessions ☐
   Strength training (e.g. lifting weights) ☐
   Cardiovascular training (e.g. running) ☐
   Cross training ☐
   Flexibility training (e.g. yoga) ☐
   Water-based training (e.g. swimming) ☐
   None ☐

4. Which emotions have you experienced in the past 24 hours?
   Joy ☐ Fear ☐ Disgust ☐ Anger ☐
   Anticipation ☐ Sadness ☐ Surprise ☐ Trust ☐
   Other (please specify) ☐ ______________________________

For Questions 5-10, please tick only **one** box.

5. How much are you looking forward to **playing** in this sports match?
   Not at all ☐ Somewhat ☐ Moderately ☐ Very much ☐

6. What have you eaten in the last two hours?
   Nothing ☐ Small snack (e.g. piece of fruit) ☐ Larger snack (e.g. toast) ☐ Full meal (e.g. pasta) ☐

7. How much are you looking forward to the **exercise** involved in this sports match?
   Not at all ☐ Somewhat ☐ Moderately ☐ Very much ☐

8. How much water have you drunk today?
   None ☐ 0-0.5L ☐ 0.5-1L ☐ 1L+ ☐

9. How motivated do you feel to win this sports match?
   Not at all ☐ Somewhat ☐ Moderately ☐ Very much ☐

To thank you for your time taken participating in this study, we will provide you with a snack whilst you complete the task after the match. Please indicate below which item of food you would prefer.

None ☐ Apple☐ Banana ☐ Orange ☐
