# Supplementary material for: The influence of supraliminal priming on energy density of food selection: a randomised control trial
Source: BMC Psychol. 2021 Mar 23;9:48. doi: 10.1186/s40359-021-00554-1 (PMC7988930; doi:10.1186/s40359-021-00554-1)
Supplement: Supplementary file 5 — Additional file 5. Post-match Questionnaire, Participant Post-match Questionnaire, questionnaire completed by all participants immediately after sports match. [file 40359_2021_554_MOESM5_ESM.pdf]

Participant Post-Match Questionnaire

You are being invited to participate in a research study titled: *The life of an athlete at university.* This study is being done by Isabelle Schlegel from the University of St Andrews. The purpose of this research study is to explore the impact of the team sport a student athlete plays (to a competitive level) on their physical, psychological and social wellbeing, including: sleep, nutrition, social life and academic work. This questionnaire will take you approximately 5 minutes to complete. Your participation in this study is entirely voluntary and you can withdraw at any time. You are free to omit any question.

Full Name:

____

Section 1

1. How tired do you feel currently? (BORG Perceived Exertion Scale: 6 = no exertion 20 = maximal exertion)

   ____

2. Have you already had something to eat or drink since the match finished? If so, what did you have?

   ____

For Questions 3-8, please tick only **one** box.

3. How much did you enjoy **playing** in this sports match?
   Not at all ☐ Somewhat ☐ Moderately ☐ Very much ☐

4. Did the score affect your enjoyment of the match?
   Not at all ☐ Somewhat ☐ Moderately ☐ Very much ☐

5. How much did you enjoy the **exercise** involved in this sports match?
   Not at all ☐ Somewhat ☐ Moderately ☐ Very much ☐

6. How much water did you drink during the match? (warm-up, pitch time, breaks and cool-down)
   None ☐ 0-0.5L ☐ 0.5-1L ☐ 1L+ ☐

7. How motivated do you feel to win your next sports match?
   Not at all ☐ Somewhat ☐ Moderately ☐ Very much ☐

8. How strong is your appetite currently? Please circle your answer. 0 = not hungry at all, 10 = I have never been more hungry

0	1	2	3	4	5	6	7	8	9	10

For Questions 9 and 10, tick **all** boxes that apply.

9. Which elements of your fitness did you utilise during the sports match?
   - Cardiovascular endurance ☐
   - Muscular strength ☐
   - Muscular endurance ☐
   - Power ☐
   - Speed ☐
   - Agility ☐
   - Flexibility ☐
   - Balance and co-ordination ☐
   - Motor skills ☐
   - Body composition ☐
   - None ☐

10. Which emotions did you experience during the match? (warm-up, pitch time, breaks and cool-down)
    - Joy ☐
    - Anger ☐
    - Fear ☐
    - Trust ☐
    - Disgust ☐
    - Sadness ☐
    - Anticipation ☐
    - Surprise ☐
    - Other (please specify) ☐ ______________________________

Section 2

11. On a scale of 6-20 how would you rate the perceived exertion of the athlete in Picture A? (6 = no exertion 20 = maximal exertion)

12. On a scale of 6-20 how would you rate the perceived exertion of the athlete in Picture B? (6 = no exertion 20 = maximal exertion)

13. On a scale of 6-20 how would you rate the perceived exertion of the athlete in Picture C? (6 = no exertion 20 = maximal exertion)

14. On a scale of 6-20 how would you rate the perceived exertion of the athlete in Picture D? (6 = no exertion 20 = maximal exertion)

15. What did you think this study was about?

    ____
